# Comparison of clustering and phenotyping approaches for subclassification of type 2 diabetes and its association with remission in Indian population

**DOI:** 10.1038/s41598-024-71126-7

**Published:** 2024-08-31

**Authors:** Pramod Tripathi, Anagha Vyawahare, Nidhi Kadam, Diptika Tiwari, Mayurika Das Biswas, Thejas Kathrikolly, Baby Sharma, Venugopal Vijayakumar, Maheshkumar Kuppusamy

**Affiliations:** 1 Freedom From Diabetes Research Foundation, Parth, Ghodke Chowk, Prabhat Rd, Deccan Gymkhana, Pune, Maharashtra 411004 India; 2 Government Yoga and Naturopathy Medical College & Hospital, Arumbakkam, Chennai, Tamilnadu 600106 India

**Keywords:** Endocrinology, Health care, Medical research

## Abstract

Identification of novel subgroups of type 2 diabetes (T2D) has helped improve its management. Most classification techniques focus on clustering or subphenotyping but not on both. This study aimed to compare both these methods and examine the rate of T2D remission in these subgroups in the Indian population. K-means clustering (using age at onset, HbA1C, BMI, HOMA2 IR and HOMA2%B) and subphenotyping (using homeostatic model assessment (HOMA) estimates) analysis was done on the baseline data of 281 patients with recently diagnosed T2D who participated in a 1-year online diabetes management program. Cluster analysis revealed three distinct clusters: severe insulin-deficient diabetes (SIDD), severe insulin-resistant diabetes (SIRD), and mild obesity-related diabetes (MOD) while subphenotyping showed four distinct categories: hyperinsulinemic, insulinopenic, classical, and nascent T2D. Comparison of the two approaches revealed that the clusters aligned with phenotypes based on shared characteristics of insulin sensitivity (IS) and beta cell function (BCF). Clustering correctly identified individuals in nascent group (high IS and BCF) as having mild obesity related diabetes which subphenotyping did not. Post-one-year intervention, higher remission rates were observed in the MOD cluster (*p* = 0.383) and the nascent phenotype showing high IS and BCF (*p* = 0.061, Chi-Square test). In conclusion, clustering based on a comprehensive set of parameters appears to be a superior method for classifying T2D compared with pathophysiological subphenotyping. Personalized interventions may be highly effective for newly diagnosed individuals with high IS and BCF and may result in higher remission rates in these individuals. Further large-scale studies are required to validate these findings.

## Introduction

Approximately 537 million adults around the world suffer from diabetes, with over 90% of these individuals having type 2 diabetes (T2D)^1^. A deeper understanding of the aetiopathogenesis of T2D may help arrest the progression of the disease and prevent diabetes-related complications. It is crucial to emphasize the clinical significance of identifying individuals at higher risk of developing diabetes complications, as this can provide valuable insights into the underlying pathological abnormalities and guide the selection of appropriate treatments^2^. By subclassifying T2D, healthcare providers may better manage the condition and offer more personalized treatment options for patients^2–4^.

Subclassifications based on phenotypes and genotypes have been reported previously. Studies in the Swedish, Danish, and Indian populations have classified T2D using either clustering or subphenotyping^3–5^. The clustering approach has resulted in the identification of several subgroups, including severe insulin-deficient diabetes (SIDD), severe insulin-resistant diabetes (SIRD), mild obesity-related diabetes (MOD), mild age-related diabetes (MARD), and one novel cluster reported in the Indian population, combined insulin-resistant and insulin-deficient diabetes (CIRDD)^3^. The second method which is subphenotyping uses homeostatic model assessment (HOMA) for insulin sensitivity (IS) and beta cell function (BCF) because T2D is primarily characterised by beta cell dysfunction, insulin resistance, or a combination of both^6,7^. Consequently, subphenotype classification based on the factors determining insulin kinetics holds greater potential for delivering individualised treatment to T2D patients with improved efficacy. In the Danish population, a phenotypic classification system based solely on BCF and IS has identified three predominant subtypes: hyperinsulinemic, insulinopenic, and classical T2D^5^.

The importance of subclassifying diabetes has been highlighted in previous studies. For instance, the SIDD cluster has the highest risk of early retinopathy and neuropathy. Compared to patients in the MARD cluster, those in the SIRD cluster were five times more likely to develop chronic kidney disease (CKD) and end-stage renal disease (ESRD)^4^. Thus, subclassification may offer an opportunity for individualised treatment and better management of T2D.

Furthermore, T2D remission has become a crucial aspect of disease management^8^. The Association of British Clinical Diabetologists (ABCD) and the Primary Care Diabetes Society (PCDS) acknowledge the possibility of achieving T2D remission, provided beta cells function properly^9^. A comprehensive subclassification offers a deeper understanding of BCF and insulin sensitivity in individual patients as well as their potential for achieving T2D remission. Previous studies have reported the risk factors associated with these subgroups. Currently, no data are available on T2D remission based on the subclassification of T2D. Furthermore, most existing subclassifications have predominantly focused on either clustering or pathophysiological phenotyping but rarely on both. Therefore, our primary aim was to compare the two classification systems and examine the rate of T2D remission in these subgroups in the Indian population.

## Methods

### Study cohort

The current study was based on data from Freedom from diabetes clinic running an online diabetes management program. Clustering and subphenotyping were performed on retrospectively extracted baseline data, and remission was reported after one year of intensive lifestyle intervention. The eligibility criteria were recent diagnosis of T2D (based on WHO criteria^10^), less than two years since diagnosis, availability of data on parameters used for classification (body mass index (BMI), glycated haemoglobin (HbA1C), plasma glucose levels, and fasting C-peptide), and data on endline HbA1C to define remission post-one-year intervention.

**Fig. 1:**
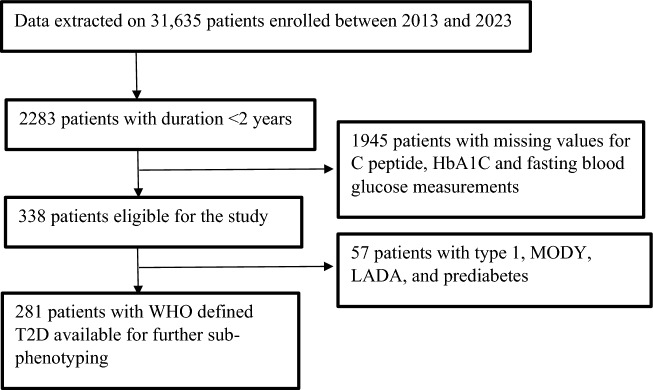
Study flow chart.

The patient flow is described in Fig. 1. Based on the availability of data on biochemical parameters for classification, a total of 281 T2D patients were included in the final analysis for subphenotyping and cluster analysis. All biochemical tests were performed at the National Accreditation Board for Testing and Calibration Laboratories (Government of India)-accredited laboratories and submitted by the patients during each consultation. The study adhered to ethical guidelines and all procedures involving human participants were conducted in accordance with the Declaration of Helsinki. Approval for this study was obtained from the institutional ethics committee of Dr. D. Y. Patil Vidyapeeth, Pune (DYPV/EC/138/16).

### Subphenotyping

**Fig. 2:**
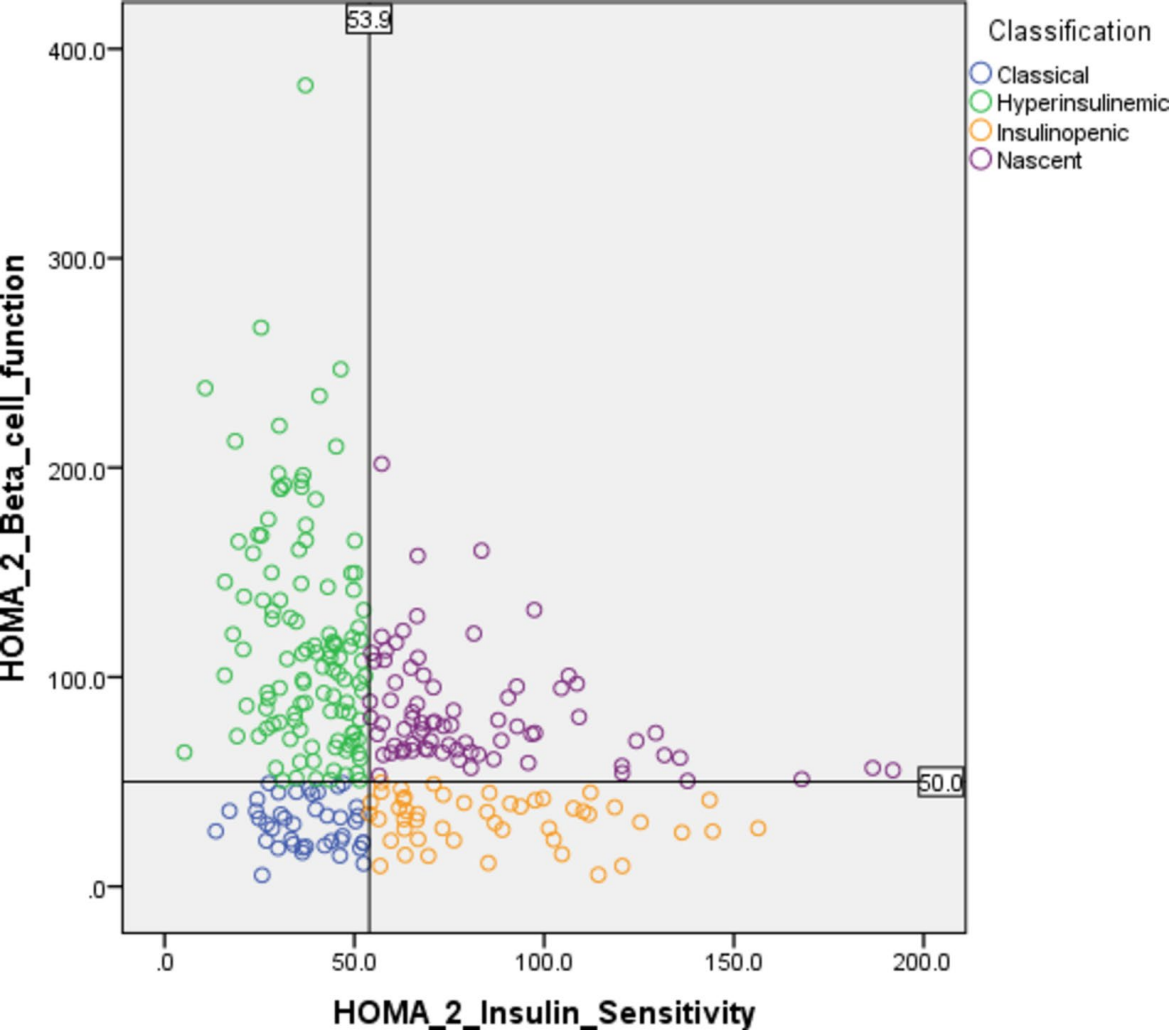
Plot of insulin sensitivity and beta-cell function. The lines mark the distinction between the four phenotypes: hyperinsulinemic, insulinopenic, classical, and nascent type 2 diabetes; the colours mark the four subphenotypes identified in the main analysis; the values used as cutoff are from Indian non-diabetic population^11,12^.

Homeostatic model assessment (HOMA) was used to categorise patients into different subphenotypes using methods described previously^5^. We used version 2 of the revised homeostatic model assessment (HOMA2 calculator) to estimate insulin sensitivity (HOMA2 IS) and beta cell function (HOMA2%B) based on fasting C-peptide and fasting plasma glucose values^11^. High and low values of IS and BCF were defined based on the median values of HOMA2 IS (53.9) and HOMA2%B (50) in a nondiabetic Indian population^12,13^. For each patient, HOMA2%B (Y‐axis) was plotted against HOMA2 IS (X‐axis) and using the cut-offs for HOMA2 IS and HOMA2%B, the data were classified into four subphenotypes: classical (low IS and low BCF), hyperinsulinemic (low IS and high BCF), insulinopenic (high IS and low BCF), and nascent (high IS and high BCF) (Fig. 2).

### Cluster analysis

We applied the k-means clustering method using the k-means function (max iteration = 1000) and the same five variables (age at onset, BMI, HbA1C, HOMA2 IR and HOMA2%B) as reported previously^2^. The optimal number of clusters was evaluated using the silhouette method which is considered the most objective method^2,3,7,14^ (Supplementary Figure S1). To test the robustness of the average silhouette method, we applied an alternative: the elbow method^3,7^ (Supplementary Figure S2). The decision tree model was used to evaluate the effectiveness of the clustering methodology by training it on the dataset, with the variables used for clustering as features and the cluster labels as the target variable. The reported accuracy of 93% reflects the ability of the model to predict the assigned cluster labels based on the input features, serving as an internal validation of the robustness of the clustering approach for accurately categorising the data points into their respective clusters. As this study employed unsupervised clustering, true labels for comparison were not available; thus, the decision tree model was utilised for internal validation rather than comparison against external true labels. The cluster-forming tendency of the data was validated using a Hopkins statistical value^3,7^. The Hopkins statistic value of 0.2 suggests that there was a moderate clustering tendency in the dataset. Cluster-wise stability was computed using the Jaccard bootstrap method by resampling the dataset 2000 times; a stable cluster should yield a Jaccard similarity index greater than 0.75^3,7,15^ (Supplementary Table S1). Cluster analysis was performed on the scaled and centred values. Cluster labels were assigned based on the phenotypic characteristics of individual cluster mean values of variables from previously published studies^2,3^.

### Replication of Swedish clusters

Furthermore, we attempted to replicate the clusters identified in the Swedish population since we used the same variables used by them (age at onset, HbA1C, BMI, HOMA2 IR, and HOMA2%B)^2^. We used k-means clustering (k = 4) and analysed the silhouette score which was 0.35 (Supplementary Figure S1). Cluster-wise stability was computed using the Jaccard bootstrap method by resampling the dataset 2000 times (Supplementary Table S2).

### Patient flow across the clusters and subphenotypes

We used a Sankey diagram to compare patient flow between the three identified clusters and four phenotypes.

### Intervention

The 1-year online intensive lifestyle intervention comprised four integrated protocols: diet, exercise, psychological support, and medical management. The details of the protocol have been described previously^16^. Diet modifications included a customized plant-based diet, intermittent fasting for weight loss, and increased protein intake for muscle strengthening, which was introduced phasewise. The exercise protocol was focused on increasing and maintaining strength, flexibility, and stamina. Psychological support included group therapy focused on relieving stress and anxiety and improving the overall mental health of patients. Medical management included daily monitoring and drug dose adjustments by a physician through a dedicated mobile application, along with supplementation for micronutrient deficiencies. The primary mode of delivery of the intervention was online through video meetings, conferences, and group sessions.

Patients were advised to seek individual medical consultation once every three months to monitor their progress. Anthropometric and biochemical parameters were collected during the follow-up visits. Regular monitoring with monthly follow-up calls, 12 live monthly group sessions, and recorded exercise and recipe videos was performed to encourage adherence to the protocol. The total program duration was one year. Post-intervention remission was defined as maintaining an HbA1C level < 48 mmol/mol for at least 3 months without the use of any glucose-lowering medications^17^.

### Statistical analyses

Statistical analyses were performed using IBM SPSS ver. 21 and Python (V.3.8). Within each group, the median (interquartile range) or mean ± standard deviation was reported based on data distribution. The significance of the difference between the group means for age at onset, BMI, fasting blood glucose, lipid profile, C-peptide, HOMA2 IR, HOMA2%B, and HbA1C was tested using the Kruskal–Wallis test (between more than two groups) and Mann‒Whitney U test (between two groups). The Chi-Square test was used to examine the associations between categorical variables. Statistical significance was set at *p*-value < 0.05.

## Results

At the time of enrolment, 65.6% of patients were on glucose-lowering medication, 27.0% were drug-naïve, and 7.4% used insulin in combination with glucose-lowering medication (oral hypoglycaemic agents). The median time from diagnosis to enrolment was 449 days [IQR 157–741 days]. Among the 281 newly diagnosed T2D patients eligible for further phenotyping and clustering, the mean age was 42.3 ± 11.3 years, and 59.4% were male.

### Subphenotyping

Figure 2 shows four distinct subphenotypes classified based on median values for IS and BCF in the non-diabetic Indian population as cutoffs^12,13^. Group 1 (lower right) was characterised by normal to high IS but severely reduced BCF (insulinopenic T2D) and accounted for 17.0% of the patients. Group 2 was characterised by low IS and reduced BCF (classical T2D), accounting for 14.6% of the patients. Group 3 was characterised by low IS but normal to high BCF (hyperinsulinemic T2D) and accounted for 40.9% of the patients. The fourth group was characterised by both high IS and high BCF (nascent T2D) and accounted for 27.4% of the patients.

Table 1 shows a comparison of anthropometric, biochemical, and medical parameters among the four subphenotypes. Compared with other subphenotypes, hyperinsulinemic patients showed the highest BMI (median 29.0 kg/m^2^) (Kruskal Wallis test, *p* < 0.001), statin medication use (Chi-Square test, *p* = 0.800), anti-hypertensive medication use (Chi-Square test, *p* = 0.012), and history of heart disease, with a 30.4% remission rate (Chi-Square test, *p* = 0.383). Among all other categories, the classical T2D group showed a higher number of patients on insulin in combination with glucose-lowering medication, with significantly higher HbA1C (Kruskal Wallis test, *p* = 0.001), abnormal lipid profile (Kruskal Wallis test, *p* < 0.05), and the second highest use of statins (Chi-Square test, *p* = 0.800). The insulinopenic group showed the lowest use of statins and the highest prevalence of substance use with a 35.4% remission rate, although the difference was not statistically significant (Chi-Square test, *p* = 0.383).

**Table 1:** Comparison of anthropometric, biochemical, and medical characteristics in the four identified Subphenotypes.

Parameter	Hyperinsulinemic (n = 115)	Insulinopenic (n = 48)	Classical (n = 41)	Nascent (n = 77)	*P* value
Age at onset (years)	44.3 ± 12.1	41.9 ± 9.6	39.8 ± 11.2	40.9 ± 10.9	0.098
Gender: Male	59 (51.3%)	29 (60.4%)	28 (68.3%)	51 (66.2%)	0.112
HOMA2 IS	37.2 (20.6–53.8)	81.9 (38.2–125.8)^a^	37.2 (19.9–54.5)^b^	70.8 (40.8–100.8)^a, c^	< 0.001
HOMA2%B	108.6 (40.5–176.7)	34.5 (19.6–49.4)^a^	29.8 (12.8–46.8)^a, b^	76.4 (44.8–108)^a, b, c^	< 0.001
HOMA2 IR	2.7 (1.5–3.8)	1.2 (0.6–1.8)^a^	2.7 (1.4–3.9)^b^	1.4 (0.9–1.9)^a, c^	< 0.001
HbA1C (mmol/mol)	52.4 (33.8–71)	66.7 (29.5–104.0)^a^	91.8 (42.7–140.9)^a, b^	45.9 (28.4–63.4)^a, b, c^	< 0.001
BMI (kg/m^2^)	29.0 (21.6–36.4)	23.3 (17.4–29.2)^a^	25.6 (17.5–33.7)^a, b^	24.4 (20.1–28.6)^a^	< 0.001
Fasting blood glucose (mmol/L)	6.6 (4.5–8.7)	9.0 (6.5–11.5)^a^	13.2 (8.7–17.7)^a, b^	5.9 (4.8–7.0)^a, b, c^	< 0.001
Total Cholesterol (mg/dL)	167.5 (114.3–220.7)	190.0 (117.0 -266.0) ^a^	201.5 (160.7–242.8)^a^	171.0 (115.3–226.7)^b, c^	< 0.001
HDL (mg/dL)	38.0 (24.0–42.0)	42.0 (27.6–56.4)^a^	39.2 (29.6–48.8)	43.4 (29.9–56.9)^a^	0.013
Triglyceride (mg/dL)	130.5 (43.8–217.2)	110.0 (37.9–182.1)	170.5 (21.5–320.3)^a, b^	112.2 (49.3–175.1)^a, c^	< 0.001
LDL (mg/dL)	104.9 (57.2–152.6)	120.0 (63.0–177.0)^a^	131.5 (91.7–171.3)^a^	107.0 (58.0–156.0)^b, c^	< 0.001
On statins	48 (41.7%)	16 (33.3%)	16 (39.0%)	30 (38.9%)	0.800
On anti-hypertensive medication	39 (33.9%)	9 (18.8%)	7 (17.1%)^a^	12 (15.6%)^a^	0.012
On heart medicine	5 (4.3%)	3 (6.3%)	-	1 (1.3%)	0.243
On OHAs	68 (59.1%)	33 (68.8%)	33 (80.5%)^a^	52 (67.5%)	0.022
On both OHAs & insulin	6 (5.2%)	5 (10.4%)	5 (12.2%)	5 (6.5%)	
Drug Naïve	41 (35.7%)	10 (20.8%)	3 (7.3%)^a^	20 (25.9%)	
Substance use*	22 (19.1%)	12 (25.0%)	10 (24.4%)	14 (18.2%)	0.717
Remission rate	35 (30.4%)	17 (35.4%)	12 (29.2%)	32 (41.5%)	0.383

Data for all parameters are presented as mean ± standard deviation or median (interquartile range) or frequency (%); BMI, body mass index; HbA1C, glycated haemoglobin; HDL, high-density lipoprotein; LDL, low-density lipoprotein; OHAs, oral hypoglycemic agents.

*Smoking or alcohol or tobacco or a combination of any two; Kruskal‒Wallis test used to compare difference in mean between more than one group; Significance of difference between categorical variables was tested using Chi-Square test; Mann Whitney U test was used to test significance of the difference in means between 2 groups.

^a^ significantly different from hyperinsulinemic.

^b^ significantly different from insulinopenic; ^c^ significantly different from classical.

### Cluster analysis

Post cluster analysis, based on the silhouette score of 0.4, we considered a k value of 3 to be the optimal number of clusters with the following distributions: severe insulin-deficient diabetes (SIDD) (34.5%), severe insulin-resistant diabetes (SIRD) (13.5%), and mild obesity-related diabetes (MOD) (52%) (Table 2).

**Table 2:** Comparison of anthropometric, biochemical, and medical characteristics in the three optimal k-value clusters.

Parameter	SIDD (n = 97)	SIRD (n = 38)	MOD (n = 146)	*P* value
**Age at onset (years)**	**40.4 ± 10.6**	**43.6 ± 12.4**	**43.2 ± 11.3**	**0.179**
Gender: Male	65 (67.0%)	20 (52.6%)	82 (56.1%)	0.158
**BMI (kg/m**^**2**^**)**	**24.5 (17.3–31.7)**	**31.7 (23.4–40.0)**^a^	**25.9 (18.3–33.5)**^a, b^	** < 0.001**
**HbA1C (mmol/mol)**	**84.2 (39.9–128.5)**	**43.7 (31.7–55.7)**^a^	**51.4 (35.0–67.8)**^a, b^	** < 0.001**
**HOMA2%B (%)**	**33.8 (12.8–54.8)**	**170.3 (121.9–218.7)**^a^	**81.8 (44.5–119.1)**^a, b^	** < 0.001**
**HOMA2 IR**	**1.8 (0.4–3.2)**	**2.8 (1.2–4.4)**^a^	**1.9 (0.9–2.9)**^**b**^	** < 0.001**
C peptide (nmol/L)	0.6 (0.2–1.0)	1.3 (0.7–1.9)^a^	0.8 (0.4–1.2)^a, b^	< 0.001
Fasting blood glucose (mmol/L)	10.3 (4.8–15.8)	5.3 (4.1–6.5)^a^	6.4 (4.9–7.9)^a, b^	< 0.001
Total Cholesterol (mg/dL)	192.0 (138.7–245.3)	169.1 (118.5–219.7)^a^	170.0 (114.6–225.4)^a^	< 0.001
HDL (mg/dL)	40.0 (28.3–51.7)	37.8 (24.0–51.6)	40 (26.8–53.2)	0.486
Triglyceride (mg/dL)	134.6 (27.1–243.9)	129.7 (48.6–210.8)	120.0 (39.9–200.1)^a^	0.072
LDL (mg/dL)	124.0 (75.0–173.0)	104.2 (55.6–152.8)^a^	107.0 (58.9–155.1)^a^	< 0.001
On statins	34 (35.1%)	17 (44.7%)	59 (40.4%)	0.527
On anti-hypertensive medication	17 (17.5%)	18 (47.3%)^a^	32 (21.9%)^a, b^	0.001
On heart medicine	2 (2.1%)	3 (7.8%)	4 (2.7%)	0.201
On OHAs	74 (76.3%)	23 (60.5%)^a^	89 (60.5%)^a^	< 0.001
On both OHAs & insulin	12 (12.4%)	1 (2.6%)	8 (5.5%)	
Drug Naïve	11 (11.3%)	14 (36.8%)	49 (33.6%)	
Substance use*	26 (26.8%)	8 (15.8%)	26 (17.8%)	0.173
Remission rate	28 (28.9%)	9 (23.7%)	59 (40.4%)	0.061

Parameters in bold are used for clustering; data for all parameters are presented as mean ± standard deviation or median (interquartile range) or frequency (%); BMI, body mass index; HbA1C, glycated hemoglobin; HOMA2 IR, homeostatic model assessment of insulin resistance; HOMA2%B, homeostatic model assessment of beta-cell function (HDL), high-density lipoprotein; LDL, low-density lipoprotein; OHAs, oral hypoglycemic agents.

*Smoking or alcohol or tobacco or a combination of any two; Kruskal‒Wallis test used to compare difference in mean between more than one group; Significance of difference between categorical variables was tested using Chi-Square test; Mann Whitney U test was used to test significance of the difference in means between 2 groups.

^a^ significantly different from SIDD.

^b^ significantly different from SIRD.

The characteristics of the three clusters were examined (Table 2). The SIDD cluster was characterised by higher HbA1C and fasting blood glucose levels, and lower BMI, HOMA2 IR, HOMA2%B, and C-peptide levels compared to both the SIRD and MOD groups, respectively, as tested by the Mann–Whitney U test (*p* < 0.05). This cluster also had significantly higher total cholesterol and LDL levels than the other clusters (Mann–Whitney U test, *p* < 0.001). The SIRD cluster was characterised by the highest BMI, C-peptide levels, HOMA2 IR, and HOMA2%B compared with the other groups (Mann–Whitney U test, *p* < 0.001). The MOD cluster was characterised by obesity but not insulin resistance (HOMA2 IR). The remission rates were highest in the MOD group, followed by the SIDD and SIRD groups, with marginal significance (*p* = 0.061, Chi-Square test).

### Replication of Swedish clusters

Forced clustering with k = 4 (Silhouette score 0.35) was performed to replicate the original Swedish clusters since we used the same parameters as used by them. The analysis showed that of the four original clusters, we could replicate three in our cohort (SIDD, SIRD, and MOD), while a fourth cluster MARD was not identified. Instead, we identified a unique cluster-combined insulin-resistant and deficient diabetes (CIRDD), previously reported in the Indian Population^3^. Higher remission rates were observed in this cluster, followed by the MOD, SIRD, and SIDD clusters, although the difference was not statistically significant (Chi-Square test, *p* = 0.367) (Supplementary Table S3). Furthermore, this cluster merged with the MOD and SIRD clusters when optimal k-means clustering was performed (Supplementary Figure S3).

### Comparison of clusters with subphenotypes

**Fig. 3:**
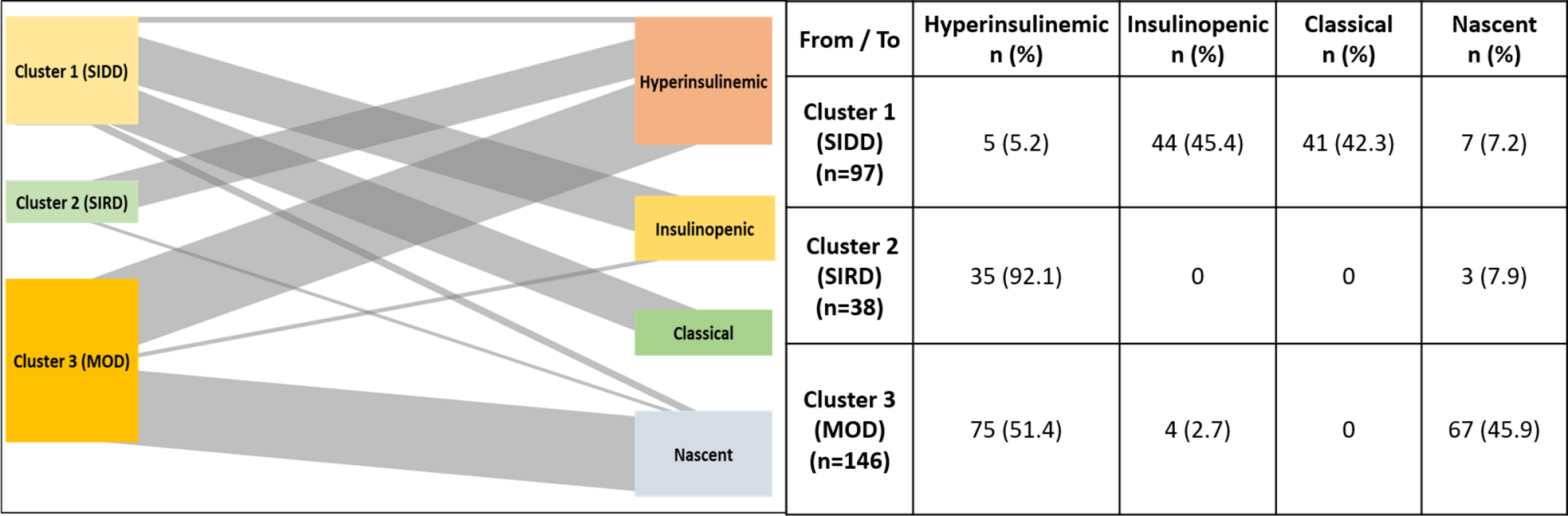
Sankey diagrams showing the flow of patients between the identified clusters and sub-phenotypes; SIDD: severe insulin-deficient diabetes; SIRD: severe insulin-resistant diabetes; MOD: mild obesity-related diabetes.

Considering the higher Silhouette score, we considered k = 3 clusters for comparison with the subphenotype classification. Since both methods used HOMA estimates as one of the components, we wanted to understand if there was any overlap between the two classifications. Initially, we used a Sankey diagram to assess the flow of patients between the two classifications. The analysis revealed that clusters merged with their respective phenotypes based on shared characteristics such as insulin sensitivity and beta cell function, clearly illustrating these associations. We observed that the SIRD cluster (92.1%) exhibited a hyperinsulinemic phenotype characterised by low insulin sensitivity i.e. insulin resistance and high beta cell function, similar to the SIRD cluster. In contrast, 45.4% and 42.3% of the patients in the SIDD cluster exhibited insulinopenic and classical phenotypes, respectively; Both phenotypes showed low beta cell function, similar to the SIDD clusters. Furthermore, 45.9% of the MOD cluster exhibited a nascent phenotype (Fig. 3). The nascent phenotype is characterised by high beta cell function and no insulin resistance (high insulin sensitivity), similar to the MOD cluster which explains the patients’ flow to the cluster.

In terms of remission, higher rates were observed in the MOD cluster, similar to the nascent phenotype. Lower remission rates were observed in the SIDD and SIRD clusters, similar to the hyperinsulinemic, insulinopenic, and classical phenotypes.

## Discussion

For the first time, pathophysiological subphenotyping was performed in an Indian population. Among 281 individuals with recently diagnosed T2D, we identified four subphenotypes (hyperinsulinemic, insulinopenic, classical, and nascent) that differed in their characteristics and rates of T2D remission. Three of these categories correspond to the subphenotypes identified in the Danish population ^5^; additionally, and we identified a category with high BCF and high IS (Nascent). This could be due to the relatively lower HOMA2%B cutoff for the Indian population, which is almost half that of the Danish population. The nascent category was excluded from the Danish study^5^; however, we chose to retain the group. As this category includes patients with both high insulin sensitivity and good beta cell function, they are likely to be in the initial stages of developing diabetes. The term "nascent” describes the early stages of diabetes development when there is both high IS and high BCF. This implies that the condition is just beginning to emerge and develop, and early intervention could potentially reverse or manage diabetes progression.

We were also able to identify three T2D clusters in the Indian population by applying data-driven cluster analysis using the key variables of age at onset, BMI, HbA1C, HOMA2 IR, and HOMA2%B used previously^2^. Post cluster analysis the distribution of variables used for clustering revealed a pattern similar to that observed in the Swedish study^2,3,7^. The three clusters identified in this study are SIDD, SIRD, and MOD. Contrary to previous findings, the MOD cluster showed the highest distribution of participants in our study cohort^2,3,18^. Furthermore, the SIRD cluster characteristics resembled those of the insulin-resistant obese diabetes (IROD) cluster identified previously in the Indian population^3^. The observation in a previous study in Swedish population that the risk of NAFLD (nonalcoholic fatty liver disease) is greater in SIRD patients than in MOD patients indicates that the severe insulin resistance observed in SIRD patients is not due to obesity alone^2^. Therefore, we preferred to use the term SIRD rather than the more generalised morphological classification of obesity in IROD^2,4,17^.

When we compared clusters with subphenotypes, clusters merged with their respective phenotypes based on shared clinical characteristics, clearly illustrating associations, contrary to a previous study where majority of the patients in SIDD and MARD cluster relocated to classical phenotype while SIRD cluster merged with hyperinsulinemic phenotype^7^. The remission rates in the clusters and phenotypes were comparable between the groups. Notably, the SIDD and SIRD clusters exhibited lower remission rates, mirroring those observed in the hyperinsulinemic, insulinopenic, and classical subphenotypes. This could be attributed to the underlying characteristics of these groups at baseline: the SIDD cluster's low beta cell function, similar to the insulinopenic and classical phenotypes, and the insulin resistance of the SIRD cluster, similar to the hyperinsulinemic phenotype, both of which contribute to their reduced likelihood of remission. It is worth discussing the nascent subphenotype observed in our cohort. This subphenotype comprised mostly of the MOD cluster and showed the lowest HbA1C and fasting blood glucose levels at baseline, similar to the MOD cluster which explains the highest remission rates among both groups. Previous studies have excluded this group from further analysis^5,7^. We chose to retain them because, despite showing high insulin sensitivity and high BCF, 74% of the patients were on either oral hypoglycaemic agents or insulin at the time of enrolment in the program and thus could not be labelled as nondiabetic. Therefore, in a clinical setting, excluding this category of patients merely based on HOMA2 IS and HOMA2%B values would be inappropriate. Therefore, even though subphenotyping offers a quick and convenient method of classifying patients in a clinical setting, special attention must be paid to those showing high IS and high BCF, who have higher chances of achieving remission.

The major strength of this study is that it reports both clustering and subphenotypic classification in the Indian population. This is the first study to report and compare T2D remission across phenotypes and clusters in the same population. One major limitation of this study is the relatively small sample size compared to other large-scale population-based studies, meaning that the findings of the study may not be applicable to the entire Indian population. Furthermore, data on additional parameters such as body composition parameters such as muscle mass, waist circumference, and waist-to-hip ratio were not available, which may have otherwise helped strengthen the findings of the study and provide further insights into the multifactorial nature of T2D. The online mode of the intervention may also have introduced variability in the adherence and the overall effectiveness of the program through variables such as access and knowledge of technology and its use, differing engagement levels, social support, and personal limitations. We employed various methods such as providing technology support for navigating the application, frequent reminders and scheduled monthly calls, feedback forms, monthly live sessions to increase engagement, personalised coaching through dedicated experts and customised interventions, and recognising and publishing success stories to encourage the participants. Furthermore, the intervention was not customised based on the subclassifications described since the classifications were performed retrospectively. Despite these limitations, our findings are clinically relevant, especially with respect to identifying the differing rates and possibility of T2D remission based on the subclassification of T2D.

In conclusion, this research highlights the importance of subclassification in the management and remission of diabetes, particularly in relation to T2D remission. It further suggests that targeted personalised interventions, may be particularly effective for individuals who are newly diagnosed with high insulin sensitivity and good beta cell function and may result in higher chances of remission. Future large-scale studies that explore the potential impact of lifestyle interventions based on T2D subclassification may provide valuable insights for the development of effective treatment plans for the management of diabetes and its associated complications.

### Supplementary Information

Supplementary Information.

## Data Availability

The data supporting the findings of this study are available from the corresponding author upon reasonable request.

## References

[1] *Atlas D. International Diabetes Federation. IDF Diabetes Atlas*. (https://diabetesatlas.org/, 2021).

[2] Ahlqvist, E. *et al.* Novel subgroups of adult-onset diabetes and their association with outcomes: A data-driven cluster analysis of six variables. *Lancet Diabetes Endocrinol.* **6**, 361–369 (2018). 29503172 10.1016/S2213-8587(18)30051-2

[3] Anjana, R. M. *et al.* Novel subgroups of type 2 diabetes and their association with microvascular outcomes in an Asian Indian population: A data-driven cluster analysis: the INSPIRED study. *BMJ Open Diabetes Res. Care* **8**, e001506 (2020). 32816869 10.1136/bmjdrc-2020-001506 PMC7437708

[4] Deutsch, A. J., Ahlqvist, E. & Udler, M. S. Phenotypic and genetic classification of diabetes. *Diabetologia* **65**, 1758–1769 (2022). 35953726 10.1007/s00125-022-05769-4 PMC9522707

[5] Stidsen, J. V. *et al.* Pathophysiology-based phenotyping in type 2 diabetes: A clinical classification tool. *Diabetes Metab. Res. Rev.* **34**, e3005 (2018). 29697198 10.1002/dmrr.3005

[6] American Diabetes Association Professional Practice Committee. 2. Classification and diagnosis of diabetes: Standards of medical care in diabetes-2022. *Diabetes Care* **45**, S17–S38 (2022). 10.2337/dc22-S002 34964875

[7] Christensen, D. H. *et al.* Type 2 diabetes classification: A data-driven cluster study of the Danish centre for strategic research in type 2 diabetes (DD2) cohort. *BMJ Open Diabetes Res. Care* **10**, e002731 (2022). 35428673 10.1136/bmjdrc-2021-002731 PMC9014045

[8] Vasdeki, D. *et al.* Remission as an emerging therapeutic target in type 2 diabetes in the era of new glucose-lowering agents: Benefits, challenges, and treatment approaches. *Nutrients* **14**, 4801 (2022). 36432488 10.3390/nu14224801 PMC9695991

[9] Nagi, D., Hambling, C. & Taylor, R. Remission of type 2 diabetes: A position statement from the Association of British Clinical Diabetologists (ABCD) and the Primary Care Diabetes Society (PCDS). *Br. J. Diabetes* **19**, 73–76 (2019). 10.15277/bjd.2019.221

[10] *WHO Classification of Diabetes Mellitus 2019*. (https://www.who.int/health-topics/diabetes#tab=tab_1, 2019).

[11] Levy, J. C., Matthews, D. R. & Hermans, M. P. Correct Homeostasis Model Assessment (HOMA) evaluation uses the computer program. *Diabetes Care* **21**, 2191–2192 (1998). 9839117 10.2337/diacare.21.12.2191

[12] Singh, Y., Garg, M. K., Tandon, N. & Marwaha, R. K. A study of insulin resistance by HOMA-IR and its cut-off value to identify metabolic syndrome in urban Indian adolescents. *J. Clin. Res. Pediatr. Endocrinol.* **5**, 245–251 (2013). 24379034 10.4274/Jcrpe.1127 PMC3890224

[13] Saha, A. Pancreatic Beta-cell function and degree of Insulin resistance among newly detected type 2 diabetics and their correlation with anthropometric, glucose, and lipid parameters: An Observational cross-sectional study. *Asian J. Med. Sci.* **13**, 71–76 (2022). 10.3126/ajms.v13i10.46707

[14] Lugner, M. *et al.* Comparison between data-driven clusters and models based on clinical features to predict outcomes in type 2 diabetes: nationwide observational study. *Diabetologia* **64**, 1973–1981 (2021). 34059937 10.1007/s00125-021-05485-5 PMC8382658

[15] Hennig, C. Cluster-wise assessment of cluster stability. *Comput. Stat. Data Anal.* **52**, 258–271 (2007). 10.1016/j.csda.2006.11.025

[16] Tripathi, P., Kadam, N., Vyawahare, A., Kuppusamy, M. & Vijayakumar, V. Long-term remission of type 2 diabetes through intense lifestyle modification program - a case series. *J. Family Med. Prim. Care* **12**, 2168–2171 (2023). 38024910 10.4103/jfmpc.jfmpc_2282_22 PMC10657089

[17] Riddle, M. C. *et al.* Consensus report: Definition and interpretation of remission in type 2 diabetes. *Diabetes Care* **44**, 2438–2444 (2021). 34462270 10.2337/dci21-0034 PMC8929179

[18] Prasad, R. B. *et al.* Subgroups of patients with young-onset type 2 diabetes in India reveal insulin deficiency as a major driver. *Diabetologia* **65**, 65–78 (2022). 34689214 10.1007/s00125-021-05543-y PMC8660725

