# Supplementary material for: Comparison of clustering and phenotyping approaches for subclassification of type 2 diabetes and its association with remission in Indian population
Source: Sci Rep. 2024 Aug 31;14:20260. doi: 10.1038/s41598-024-71126-7 (PMC11366003; doi:10.1038/s41598-024-71126-7)
Supplement: Supplementary file 1 — Supplementary Information. [file 41598_2024_71126_MOESM1_ESM.docx]

**Additional Information on cluster analysis**

**Hopkin static value** = 0.19

**Silhouette score for four Swedish replicated clusters** = 0.35

**Silhouette score for optimal k value clusters =** 0.40


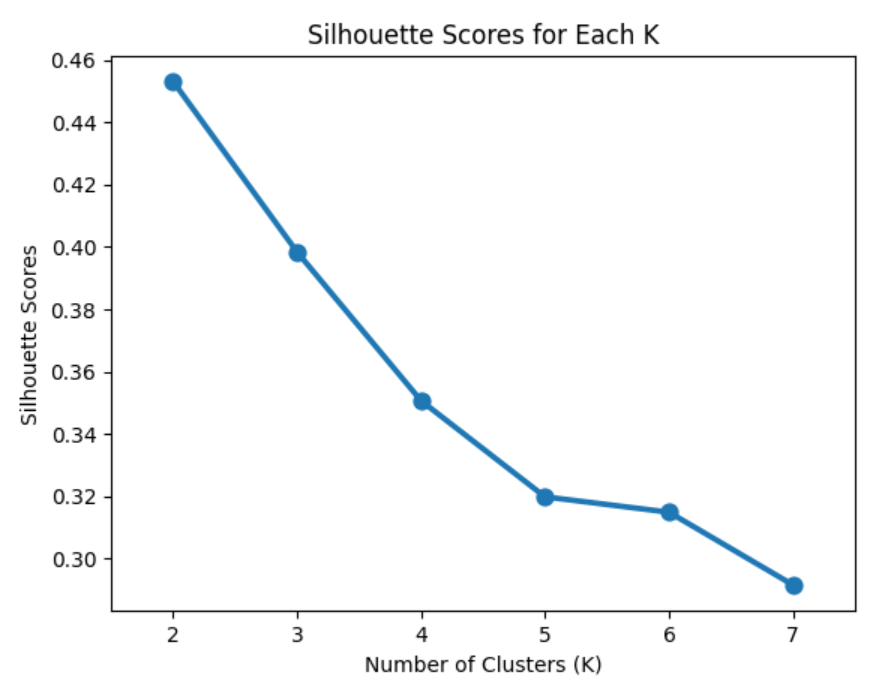


**Supplementary Figure S1: Plot of silhouette score with k value to determine optimal number of clusters**

**Elbow method**


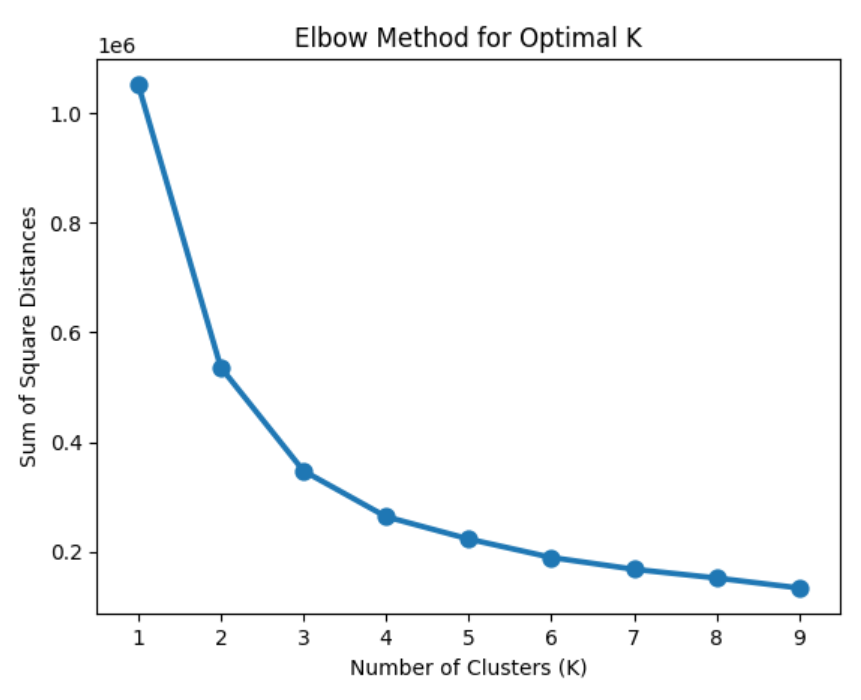


Elbow Point

**Supplementary Figure S2: Plot of sum of square distances with k value to determine optimal number of clusters**


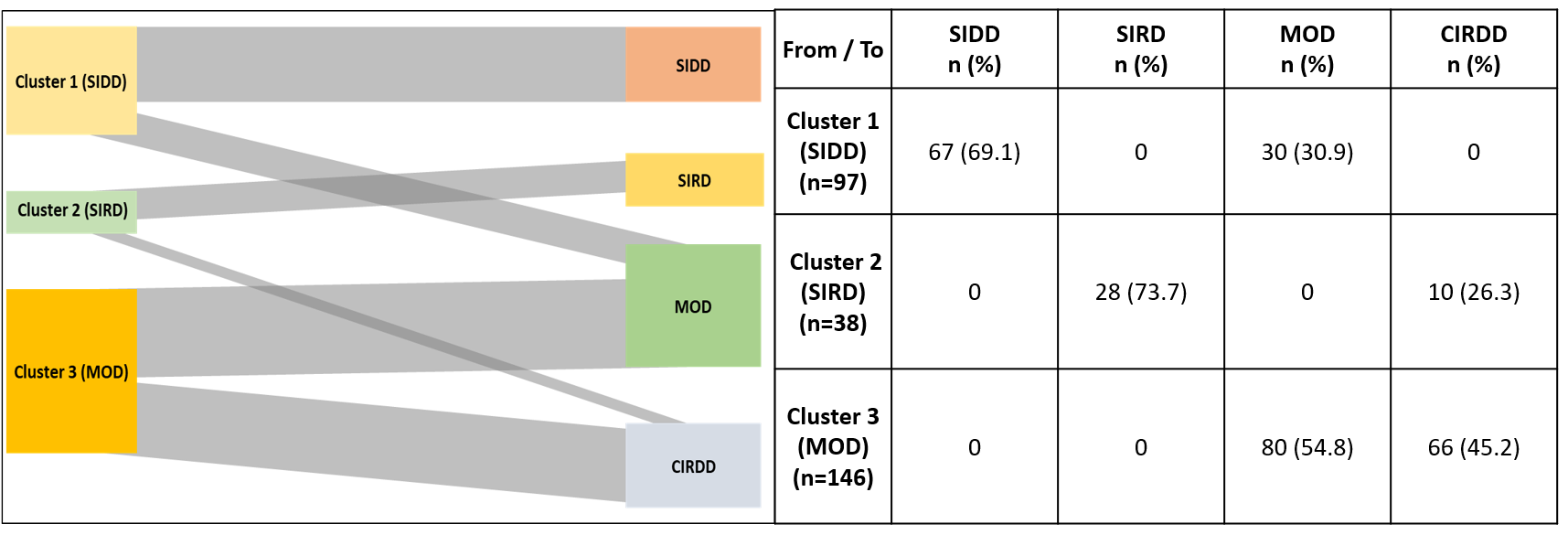


**Supplementary Figure S3:** the clusters identified using optimal k values and the clusters identified post-replicating the Swedish clusters; SIDD: severe insulin-deficient diabetes; SIRD: severe insulin-resistant diabetes; MOD: mild obesity-related diabetes; CIRDD: combined insulin-resistant and deficient diabetes

**Jaccard Similarity and distance Index**

**Supplementary Table S1: Jaccard similarity and distance index for Optimal k value clusters (3)**

| **Cluster combinations** | **Jaccard similarity index** | **Jaccard distance index** |
| --- | --- | --- |
| Cluster 1 (SIDD) & Cluster 2 (SIRD) | 0 | 1 |
| Cluster 1 (SIDD) & Cluster 3 (MOD) | 0 | 1 |
| Cluster 2 (SIRD) & Cluster 3 (MOD) | 0 | 1 |

**Supplementary Table S2: Jaccard similarity and distance index for four Swedish replicated clusters**

| **Cluster combinations** | **Jaccard similarity index** | **Jaccard distance index** |
| --- | --- | --- |
| Cluster 1 (SIDD) & Cluster 2 (SIRD) | 0 | 1 |
| Cluster 1 (SIDD) & Cluster 3 (MOD) | 0 | 1 |
| Cluster 1 (SIDD) & Cluster 4 (CIRDD) | 0 | 1 |
| Cluster 2 (SIRD) & Cluster 3 (MOD) | 0.1 | 0.9 |
| Cluster 2 (SIRD) & Cluster 4 (CIRDD) | 0.1 | 0.9 |
| Cluster 3 (MOD) & Cluster 4 (CIRDD) | 0 | 1 |

**Supplementary Table S3: Comparison of anthropometric, biochemical, and medical characteristics in the four replicated Swedish clusters**

| **Parameter** | **SIDD**  **(n = 67)** | **SIRD**  **(n = 28)** | **MOD**  **(n = 110)** | **CIRDD**  **(n = 76)** | **P value** |
| --- | --- | --- | --- | --- | --- |
| **Age at onset (years)** | **39.7 ± 9.9** | **42.4 ± 13.2** | **43.7 ± 11.3** | **42.4 ± 11.4** | **0.166** |
| Gender: Male | 48 (71.6%) | 13 (46.4%) | 53 (48.2%) | 53 (69.7%) | 0.002 |
| **BMI (kg/m^2^)** | **24.9 (17.8-32.0)** | **31.7 (22.2-41.2)** | **25.3 (18.9-31.7)** | **27.8 (19.9-35.7)** | **<0.001** |
| **HbA1C (mmol/mol)** | **99.4 (66.5-133.3)** | **43.7 (33.3-54.1)** | **55.7 (37.9-73.5)** | **50.2 (32.8-67.8)** | **<0.001** |
| **HOMA2 %B (%)** | **29.8 (12.4-47.2)** | **190.4 (144.2-236.6)** | **63.8 (38.4-89.2)** | **110.2 (85.7-134.7)** | **<0.001** |
| **HOMA2 IR** | **2 (0.6-3.4)** | **2.8 (1.4-3.2)** | **1.6 (0.6-2.6)** | **2.2 (1.2-3.2)** | **<0.001** |
| C peptide (nmol/L) | 0.7 (0.3-1.1) | 1.3 (0.8-1.8) | 0.63 (0.23-1.0) | 0.9 (0.5-1.3) | <0.001 |
| Fasting blood glucose (mmol/L) | 12.2 (7.2-17.2) | 5.1 (4.0-6.2) | 7.3 (5.4-9.2) | 6.1 (5.0-7.2) | <0.001 |
| Total Cholesterol (mg/dL) | 201.5 (147.2-255.8) | 165.6 (101.1-230.1) | 180.4 (119.9-240.9) | 161 (115.5-206.5) | <0.001 |
| HDL (mg/dL) | 39.2 (26.9-51.5) | 39.3 (22.2-56.4) | 43.4 (31.4-55.4) | 37.6 (26.6-48.6) | 0.009 |
| Triglyceride (mg/dL) | 148.0 (39.2-256.8) | 132.5 (49.4-215.6) | 115 (25.4-204.6) | 121.4 (60.4-182.4) | 0.010 |
| LDL (mg/dL) | 130.8 (87.2-174.4) | 105.1 (55.2-158.0) | 110.0 (65.8-154.2) | 99 (59.6-138.4) | <0.001 |
| On statins | 26 (38.8%) | 12 (42.8%) | 41 (37.3%) | 31 (40.8%) | 0.937 |
| On anti-hypertensive medication | 9 (13.4%) | 12 (42.8%) | 21 (19.0%) | 25 (32.9%) | 0.002 |
| On heart medicine | 5 (4.3%) | 3 (6.3%) | - | 1 (1.3%) | 0.117 |
| On OHAs  On both OHAs & insulin  Drug Naïve | 53 (79.1%)  9 (13.4%)  5 (7.5%) | 18 (64.3%)  1 (3.6%)  9 (32.1%) | 63 (57.3%)  9 (8.2%)  38 (34.5%) | 52 (68.4%)  2 (2.6%)  22 (29.0%) | 0.001 |
| Substance use* | 18 (26.9%) | 4 (14.3%) | 17 (15.5%) | 19 (25.0%) | 0.175 |
| Remission rate | 18 (26.9%) | 8 (28.6%) | 40 (36.4%) | 30 (39.5%) | 0.367 |

Parameters in bold were used for clustering; Data for all parameters are presented as mean ± standard deviation or median (Inter quartile range) or frequency (%); BMI, Body Mass Index; HbA1C, Glycated hemoglobin; HOMA2 IR, Homeostatic Model Assessment of Insulin Resistance; HOMA2 %B, Homeostatic Model Assessment of beta-cell function HDL, High density lipoprotein; LDL, Low density lipoprotein; OHAs, oral hypoglycemic agents; *Smoking or Alcohol or Tobacco or combination of any two; Comparison between groups for continuous variables was done by Kruskal Wallis test and for categorical variables by chi-square test
